# From Neural Plate to Cortical Arousal—A Neuronal Network Theory of Sleep Derived from *in Vitro* “Model” Systems for Primordial Patterns of Spontaneous Bioelectric Activity in the Vertebrate Central Nervous System

**DOI:** 10.3390/brainsci3020800

**Published:** 2013-05-22

**Authors:** Michael A. Corner

**Affiliations:** Netherlands Institute for Brain Research, Amsterdam, 1071-TC, The Netherlands; E-Mail: m.corner@hccnet.nl; Tel.: +3120-471-1977

**Keywords:** sleep ontogeny, tissue culture, multi-electrode recording, spontaneous neuronal activity, cholinergic activation, synchronized bursting, “REM-sleep” hypothesis, forebrain, hindbrain

## Abstract

In the early 1960s intrinsically generated widespread neuronal discharges were discovered to be the basis for the earliest motor behavior throughout the animal kingdom. The pattern generating system is in fact programmed into the developing nervous system, in a regionally specific manner, already at the early neural plate stage. Such rhythmically modulated phasic bursts were next discovered to be a general feature of developing neural networks and, largely on the basis of experimental interventions in cultured neural tissues, to contribute significantly to their morpho-physiological maturation. In particular, the level of spontaneous synchronized bursting is homeostatically regulated, and has the effect of constraining the development of excessive network excitability. After birth or hatching, this “slow-wave” activity pattern becomes sporadically suppressed in favor of sensory oriented “waking” behaviors better adapted to dealing with environmental contingencies. It nevertheless reappears periodically as “sleep” at several species-specific points in the diurnal/nocturnal cycle. Although this “default” behavior pattern evolves with development, its essential features are preserved throughout the life cycle, and are based upon a few simple mechanisms which can be both experimentally demonstrated and simulated by computer modeling. In contrast, a late onto- and phylogenetic aspect of sleep, viz., the intermittent “paradoxical” activation of the forebrain so as to mimic waking activity, is much less well understood as regards its contribution to brain development. Some recent findings dealing with this question by means of cholinergically induced “aroused” firing patterns in developing neocortical cell cultures, followed by quantitative electrophysiological assays of immediate and longterm sequelae, will be discussed in connection with their putative implications for sleep ontogeny.

“In biological as well as physical-chemical sciences, one should reduce phenomena to as simple as possible experimental conditions.”

—Claude Bernard [1]

## 1. Introduction

Even after more than a half-century of concerted research efforts, a number of stubborn semantic conundrums are still associated with the concept of “sleep”. An especially long-standing blind spot has been the general reluctance to appreciate the singular contributions made along the way by *in vitro* cell culture experiments in establishing a sound theoretical basis for understanding the essential features of sleep phenomenology. Upon analysis, this arbitrary cutoff line for dignifying a “model” system with the hallowed S-word turns out to be simply a semantic question: in the beginning there was only “sleep” and “wakefulness”, the former being regarded as motorically quiescent while the latter comprised diverse forms of motor activity. With the acknowledgment of motorically quiescent wakefulness as a third behavioral class, a theoretical double axis of (active *vs.* quiet) and (waking *vs.* sleeping) behavior automatically emerges. The predictable eventual filling in of the fourth, *i.e.*, “active sleep”, block of this 2 × 2 matrix was accomplished by the relatively late discovery of “rapid-eye-movment” (REM) sleep, the defining characteristic of which is the occurrence of intrinsically generated, seemingly non-purposive, poly-rhythmic motor discharges. Subsequent investigations revealed that “type IV” motility in fact comprises a broad class of behaviors—evolutionarily as well as developmentally—that fit this phenomenological description and which characterize the early ontogeny of vertebrates in general, ecto- as well as endothermic [2,3,4,5,6,7].

In the present review, applying the generally accepted basic principle of experimental biology [1], a half-century long progression of insights into the underlying neurological mechanisms of “Type IV” behavior, as attained through the study of reduced living “model systems” cultured *in vitro*, will be reviewed for both the motorically active and the quiescent phases of what is commonly known as the “Basic Rest-Activity Cycle” or “circa-horalian” (*ca.* hourly) brain sleep rhythms. “Active Sleep” strikes me as being the most (only?) appellation for this class of activity patterns (which could apply even to any as yet undiscovered physiological manifestations operating on the cellular rather than the network level—although, to tell the truth, I can imagine being reluctant on a gut level to reach that conclusion at such an extreme level of reduction; but “ye pays yer money and ye takes yer pick”, as the olde expression goes). The elegant simplicity of the 2 × 2 matrix formulation, furthermore, should also help to clear the ground of the semantic confusion and fruitless debates about what “is” or is not “sleep” that currently abound. A popularized chronological account of the progressive elaboration of the ideas presented in this review [8].

## 2. Motorically Active (“Rhombencephalic”) Sleep

**Figure 1:**
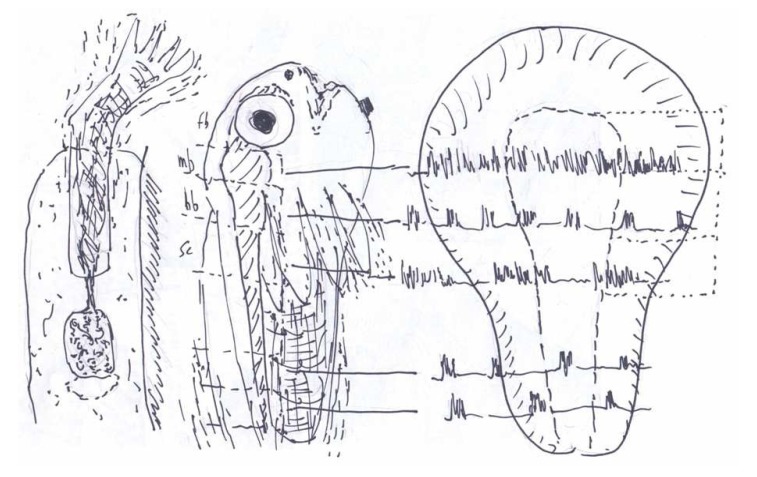
Sketch made for a lecture in 1961 at the Zoology department, Columbia University (New York City) showing: *left*—the limb/nerve deplantation experiment of Paul Weiss in the axolotl dorsal fin; *middle*—a frog tadpole showing the transections made at different CNS levels; *right*—outline of the early anuran neural plate illustrating the spontaneous motility patterns generated by isolated presumptive CNS fragments combined with mesoderm (see text and [6] for details).

Until the middle of the last century little attention was paid to the possibility of intrinsically generated neuronal activity being a ubiquitous feature of brain function and behavior. It was then reported, however, that axolotl neuromotor tissues deplanted into a dorsal fin could innervate and trigger complex movements in isolated, similarly deplanted limbs [2] (Figure 1). The paradigm shift that this discovery heralded was not widely appreciated but, at about the same time, it was discovered that “rapid eye” (REM) and other body movements occur spontaneously during sleep in humans and many other animals. This “third physiological state” turned out to be a widespread phenomenon that has its origin in neuronal discharges originating in the rostral hindbrain [3,4]. Within a decade, by taking advantage of the late development of sensory nerves in chick embryos, and subsequently employing surgical deafferentation to confirm the deduction of a “non-reflexogenic” origin within the central nervous system, the principle of spontaneous neuromotor discharges had been broadened to include the onset of motility in endothermic vertebrates [5].

**Figure 2:**
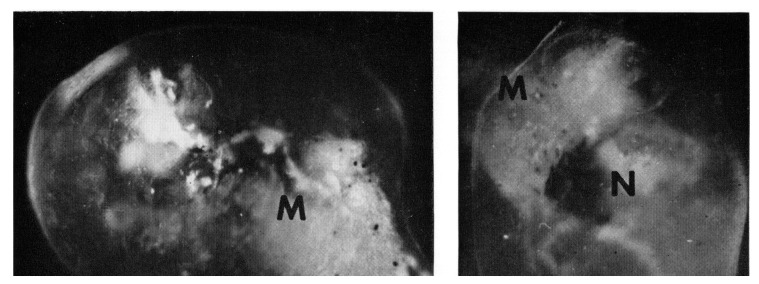
Two examples of “Frankensteinian” preparations prepared and filmed in 1961 at the Hubrecht International Embryology Laboratory in Utrecht, The Netherlands, consisting of anuran neural plate tissue, plus mesoderm, encased in a transparent ball of ectoderm. Differentiated CNS fragments (N) are covered by a pigment layer, and twitches of the muscle fibers (M) could be easily followed through a stereo microscope.

**Figure 3:**
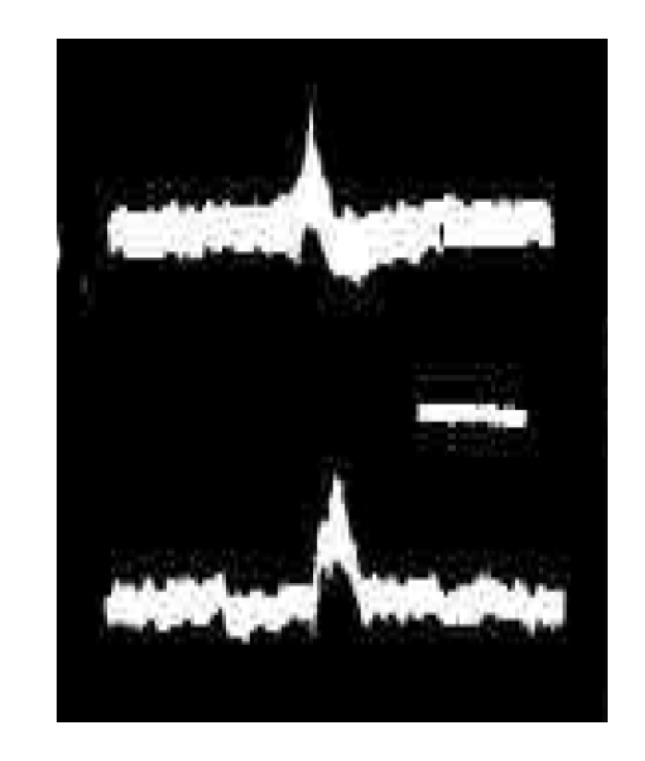
Oscillogram photographed in 1963 at Columbia University, Department of Neurology, showing the first ever example of a spontaneous polyneuronal barrage of field and action potentials (top trace) driving a stereotyped burst of muscle twitches. Recorded in an organotypic culture derived from a presumptive brainstem motor region of the frog neural plate. Time bar = 1 s.

*In vitro* culture techniques soon opened the way to an extension of this new paradigm to exothermic vertebrates [6]. Pieces of amphibian neural plate destined to become motor areas (Figure 1) could, when combined with presumptive muscle tissue and enclosed within an epithelial sheath (Figure 2), differentiate into central nervous structures that triggered spontaneous phasic contractions that were readily visible under the microscope. The addition of presumptive primary sensory neurons to these “Frankenstein models” for motorically active sleep although making possible the development of cutaneous reflex arcs, had no noticeable effect on the contractions, while presumptive forebrain areas failed altogether to support the appearance of either spontaneous or evoked twitching [7]. Indeed, the prosencephalic area of the plate was already “determined” (*i.e.*, programmed for self-differentiation) to generate only forebrain structures such as retina, tapetum and olfactory placodes [9]. Micro-electrode recordings from neural plate derived hindbrain andspinalcord cultures [10] confirmed that a stereotyped phasic episode of polyneuronal firing immediately preceded each burst of contractions (Figure 3).

**Figure 4:**
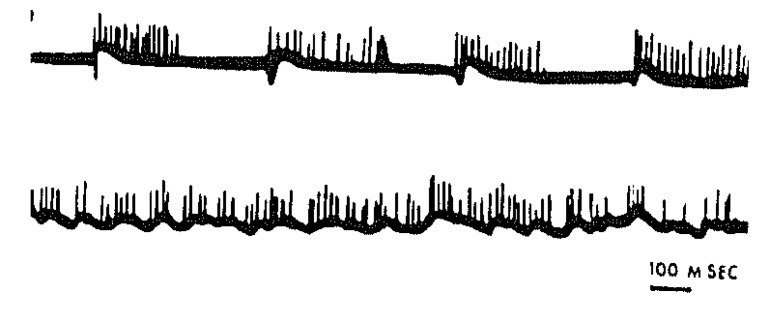
Oscillogram (slightly retouched) of stereotyped polyneuronal burst firing (upper trace) as well as continuous field potentials associated with tonic firing during an exceptionally active period in an organotypic mouse medulla culture. Recorded in 1968 at the Rose Kennedy Center, Albert Einstein College of Medicine, New York, NY, USA.

**Figure 5:**
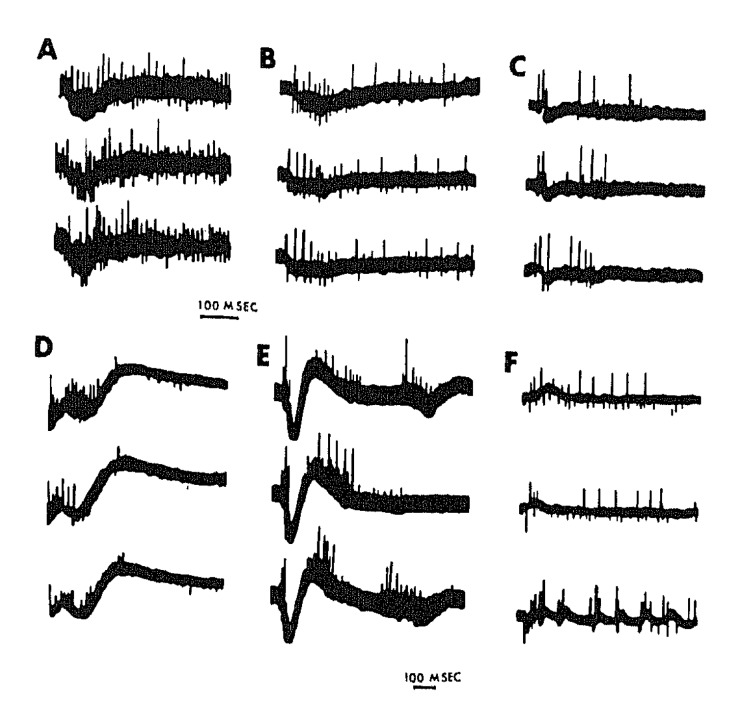
Examples of all-or-none responses triggered in different preparations or at different recording sites by electrical stimulation in organotypic mouse medulla cultures. Recorded in 1968 at the Rose Kennedy Center, Albert Einstein College of Medicine, New York, NY, USA.

Subsequent studies using organotypic mouse spinal cord or hindbrain explants [10,11] enabled this model system for stereotyped phasic discharges, including its ubiquitous fluctuations in the frequency of such discharges, to be generalized to a mammalian [12] (Figure 4). Indeed, the presence of frequency modulated, non-purposive, neurogenic bursts of spontaneous muscle contractions defines active—or “rapid-body-movement”—sleep (RBM) as a broad behavioral category in which “REM sleep” is a special case [13,14]. The threshold for electrically triggering an all-or-none discharge (Figure 5) was commonly seen to fluctuate in parallel with the level of phasic network activity, and the reappearance of stereotyped responses evoked by shocks of luminal intensity typically heralded the onset of renewed spontaneous bursting. The central origin of neuronal firing (including the ongoing “background” of irregular tonic activity, in the absence of which bursts failed to occur spontaneously: [13,15]) was established by confirming the absence of impulse traffic in functionally attached dorsal root ganglion cells. Similar behavior in dispersed neuronal network cultures indicated that structural differentiation of the spinal cord is a negligible factor in producing typically RBM-like spontaneous discharges [16], including multiple sites of origin of spreading waves of activity [17]. Apparently, almost any diffusely interconnected excitatory network will do.

Sporadic “background” firing typically appeared long before any synchronous bursts were observed and, indeed usually before the latter could be evoked by even the most intense electric shocks [18]. Only when the threshold for burst evocation had fallen to just a few volts did they start to occur spontaneously (except in a few preparations where extensive sampling failed to reveal any detectable tonic background spiking within the network). Glycinergic disinhibition greatly enhanced the amplitude—but not the duration—of the field potentials, indicating that inhibitory feedback is not the main cause of the cessation of these network bursts. A period of relative refractoriness (*i.e.*, an elevated threshold for evoking a subsequent all-or-none response) lasting a half-minute or more identified the burst frequency control mechanism as constituting an intrinsic cellular relaxation oscillation [11,15,19,20]. Since thresholds were lowered and the incidence of spontaneous bursting elevated by glycine receptor blockade [18], feed-forward synaptic inhibition is evidently also an important aspect of spontaneous activity patterns in these preparations [21].

Inhibitory drive apparently interferes with the ability of motor network bursts to consistently elicit synchronous muscle contractions—as observed in the chick embryo *in ovo* as well: [22]—since synaptic disinhibition restores the primitive strict 1:1 relation [23]. Activity-dependent inhibition also plays a crucial role in limiting the spatio-temporal recruitment of medullary and spinal cord neurons, many of which fired only if the network was pharmacologically disinhibited [15,18,24]. Despite the largely all-or-none character of synchronous network bursts (Figure 3, Figure 4 and Figure 5), a distinction can thus be made between sleep-like and paroxysmal activity on the basis of the large difference in the amplitudes of the field potentials. Fast inhibitory feedback, on the other hand, may account for the characteristic “ripples” which cause intra-burst spike trains to themselves be broken into “micro-bursts” [15,18,24].

Many deprivation experiments through the years have documented the importance of early spontaneous bioelectric activity, in the form of quasi-sleep patterns (“seismic” or “sleep-with-jerks” in the French literature: [4,25,26]), for a wide variety of downstream developmental processes [27,28]. In addition, despite an impressive degree of normal morpho-physiological maturation even in the total absence of bioelectric activity [29], a number of central effects have since been revealed using quantitative methods, perhaps the most important of which being a homeostatic up-regulation of net excitatory drive within spinal cord cultures following prolonged suppression of spontaneous spike discharges [30,31,32]. Such treatment also has been shown to lead to an abnormally diffuse, regionally non-specific, innervation of the cord explants by presented dorsal root ganglion fibers [33,34,35]. *In vitro* model systems for early sleep ontogeny thus were the first to provide evidence for the pruning of “exuberant” anatomical connections (an extremely common activity-dependent developmental phenomenon) in a target structure despite the absence of afferent stimulation. Activity dependent plastic changes such as those mentioned appear to be triggered largely by the enhanced accumulation of intracellular calcium that takes place during burst discharges [36].

A last aspect that perhaps deserves mentioning with regard to the suitability of organotypic spinal cord explants to serve as model systems for early sleep-like physiological states is their ability to be directly excited by neurologically “arousing” modulators such as acetylcholine and serotonin [37]. This would mean that mechanisms underlying the transition between sleep and wakefulness in adulthood [3,25] may in fact extend throughout the length of the lower neuraxis. There is indeed a descending rostro-caudal gradient of sleep-like spontaneous burst generation in early life [6,38,39,40], so that an ontogenetic analogy to a sinking iceberg comes to mind, where eventually only a few of the highest points eventually remain above the surface, *c.q.*, threshold. As excitability decreases under the influence of growing synaptic inhibition, the incidence of discharges will tend to stabilize into “small world” zones of weakly interacting localized activity [41]. Finally, the mentioned homeostatic compensation and “overshoot” in response to sustained alterations of spontaneous activity, whether up or down, constitutes one of the most convincing criteria for regarding early spontaneous neuromotor behavior as being a sleep state in the true sense of the word [14,16,17,18,19,20,21,22,23,24,25,26,27,28,29,30,31,32,33,34,35,36,37,38,39,40,41,42,43].

## 3. Synchronous Slow-Wave (“Telencephalic”) Sleep

**Figure 6:**
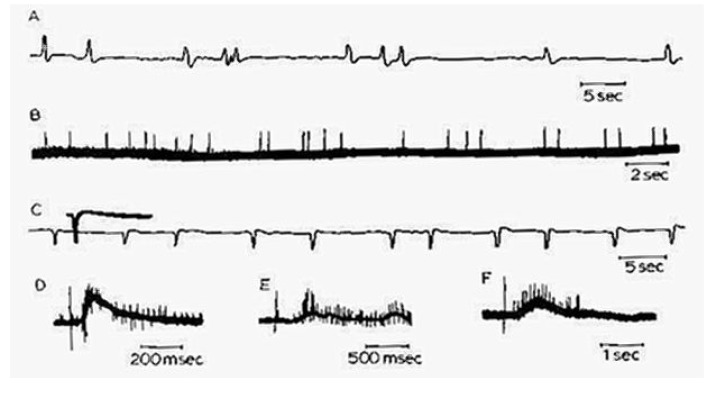
Different patterns of spontaneous field potentials or isolated spiking (**A**–**C**), and all-or-none evoked responses (**D**–**F**) in organotypic chick embryo cerebral hemisphere explants: oscillographically recorded in 1968 at the Albert Einstein College of Medicine, New York, NY, USA.

Perhaps the earliest indication that cerebral slow-wave activity during sleep has a similar neurophysiological basis as RBM at the brainstem level came from organotypic cultures of embryonic chick forebrain tissue (Figure 6). After several days of growth *in vitro*, stereotyped field potentials began to appear spontaneously every few seconds, much as they do *in ovo* under the rate-limiting control of maturing glutamic acid enzymatic systems [44,45], culminating in intrinsically generated trains of amplitude modulated slow wave complexes which typically occur in variable cycles lasting on the order of several minutes [46]. Comparable “basic waveforms” in the intact infant rat neocortex during quiet sleep were shown to be associated with discrete burst-pause neuronal discharges [47], the endogenous origin of which was first demonstrated using dispersed cortical cell cultures [48], and thereafter has been repeatedly verified using either organotypic explants [49,50,51] or dissociated neurons cultured on multi-electrode grids [52,53,54,55]. Indeed, the basic underlying mechanisms—(1) spontaneous transmitter release, (2) recurrent excitatory connectivity, and (3) activity-dependent membrane refractoriness [13]—that were discovered for the brainstem and spinal cord [11,12,13,14,16,17,18,19>] are sufficient for mimicking network bursting even in single cortical cell micro-cultures forced to intensively innervate themselves autaptically [56]. Importantly, in addition, neocortical cultures have been shown to possess several biochemical and metabolic characteristics of slow-wave sleep in the intact organism [53].

**Figure 7:**
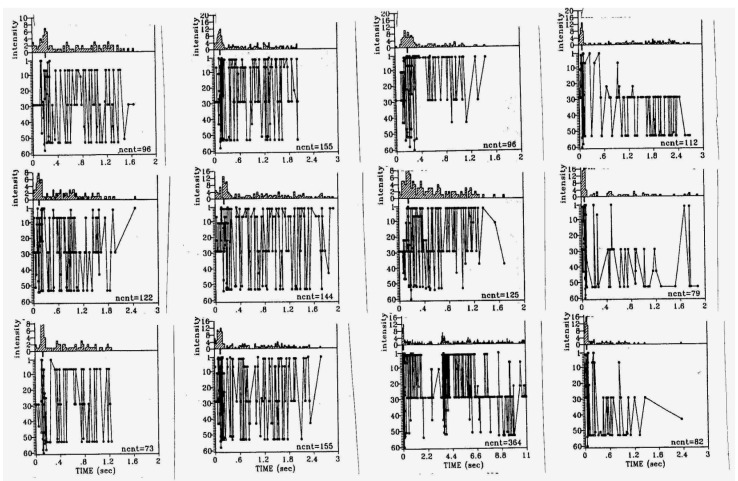
Examples of (four) different classes of more or less stereotyped neuronal firing in a dissociated rat neocortex culture recorded on a multi-electrode plate. The connecting lines indicate the sequential firing of individual spikes at the participating recording sites: experiment performed together with Dr. van Pelt, J. in 2005 at The Netherlands Institute for Brain Research, Amsterdam, The Netherlands.

High frequency short bursts of activity, such as are also seen superimposed on the “up” states of individual delta waves during SWS, and probably reflecting “flip-flop oscillations mediated by fast inhibitory synaptic feedback [25,50,57], are another feature of SWS that has been reproduced by *in vitro* models [58,59]. Although a high degree of burst reproducibility is often observed with respect to sequential action potential discharge patterns, as is also the case *in situ* during slow-wave sleep [60], the detailed patterning of polyneuronal firing appears to fall into a number of distinct classes [61,62] (Figure 7). The large degree of burst variability with respect to frequency, duration, firing intensity and regularity, within as well as between different cultures [63], is comparable to that reported for organotypic brainstem cultures [11] (Figure 4 and Figure 5). Average network firing rate profiles, on the other hand, can be highly constant for days at a time, but also show gradual developmental trends over the course of weeks [64].

Organotypic bilateral “mega-cocultures” exhibit an even greater resemblance to intact cerebral cortex function inasmuch as, independently in different locations, they generate relatively long trains of synchronous oscillatory bursts [50] which interact weakly both within and between the cultured halves [51].This preparation thus represents the *in vitro* “model system” that up till now most closely mimics normal brain wave activity at the neocortical level during sleep [60,65]. In combination with multi-electrode recording systems, it promises to make possible more realistic investigations of the formation, dynamics and interactions among “small-world” domains within the cerebral cortex [66,67,68] than would be possible using dispersed cell cultures. On the other hand, spontaneous activity even in dispersed neocortical cultures is sufficiently sleep-like—*i.e.*, characterized by periodically modulated spontaneous bursts of synchronous neuronal discharges [53,69]—that the pervasive laminar and columnar structure that distinctively characterizes this structure *in situ* must be assumed to develop primarily or exclusively with “forward reference” to the demands of sensory information processing during the waking state.

A first hint of a possible role for RBM during early cortical development came from the demonstration in organotypic cultures that, in the absence of spontaneous neuronal firing, excitatory axo-dendritic (spine) synapse numbers stabilize at an abnormally high level rather than overshooting to a lower plateau value [70,71]. Since, in contrast, excessive bursting provoked by chronic synaptic disinhibition throughout the period of early morphological development led to a premature overshoot in spine synapse formation (despite unchanged mean firing levels [72], the notion was born of a homeostatic control function for synchronous bursts of bioelectric activity in regulating the formation of excitatory connectivity [41,73]. Activity dependent homeostasis for restraining exuberant neocortical projections has indeed since been verified for *in vivo* development as well [74].

The unsuspected existence of non-Hebbian self-regulatory plasticity during early ontogeny has also been demonstrated on the basis of quantitative electrophysiological recordings but in this case it is the maturation of inhibitory synaptic drive that was first clearly implicated as a crucial activity dependent factor [71,75]. This appears to be the case *in situ* as well [76]. Many later studies have not only supported this conclusion but have demonstrated myriad additional downstream processes that, driven by self-generated neuronal firing, contribute to the homeostatic control of developing cortical excitability [77,78,79,80]. The tendency for sleep-deprived adult cortical neurons to gradually become hyperexcitable, and then slowly return to baseline levels under the influence of synchronous “slow-wave” sleep (SWS) discharges [81], seems thus to be a neotenic retention of a highly primitive form of neuroplasticity. It has been suggested that spontaneous sleep-like bursting is a naturally occurring form of post-tetanic depression, triggered by massive calcium entry during each wave [82,83], and followed by the induction of a broad spectrum of homeostatically appropriate gene activation [56,83].

The involvement of specifically glutamatergic synapses in SWS homeostasis [81] has been shown using organotypic explants to be an intrinsic neocortical phenomenon. In developing isolated explants, AMPA synaptic drive replaces NMDA when the latter are blocked, whereas when both receptor sub-types are blocked the networks remain silent but become hyperactive when returned to normal medium [41]. When, on the other hand, two cortex explants were co-cultured so as to permit mutual cross-innervation, kainate receptor drive increased during AMPA + NMDA receptor blockade enough to sustain ongoing spontaneous activity at approximately normal levels [58]. Since co-cultured cortical explants, in contrast to isolated ones, show extensive dendritic outgrowth that approximates the *in vivo* condition [84], it may be concluded that kainate receptors are situated primarily on the distal branches. Such blockade suppressed all ongoing bioelectric activity at first, but it recovered to normal levels within 24 h as the kainate receptors took over from the blocked receptors. The latter, too, may have up-regulated functionally during the period of reduced activity, since a sizeable transient overshoot occurred when the cultures were returned to normal medium [69].

Renewed AMPA/NMDA blockade, however, led to the now exclusively kainate driven bursts causing the blocked receptors to gradually become desensitized to the point that they no longer contribute significantly to excitatory drive upon return to normal medium [58]. This compound sequence of homeostatic adjustments in cultured cortical networks is precisely what one would expect from a “model” for plasticity mechanisms during SWS. The unexpected versatility of receptors in taking over from one another—even total glutamatergic blockade fails to permanently eliminate spontaneous bursting, since intrinsic cholinergic neurons assume an overtly excitatory role under those conditions [59]—is a fascinating property of this model system that would repay investigation in whole animal studies of sleep.

## 4. Cholinergic “Arousal”: Acute and Long-Lasting Effects on Neocortical Function and Plasticity

An important aspect of neocortical sleep maturation is the phylo- [4] and ontogenetically [85] late developing link to the brainstem arousal system, whereby forebrain slow-wave activity becomes “paradoxically” desynchronized into a quasi-aroused mode of neuronal firing [3,14,26]. This activity has been hypothesized to provide a needed endogenous source of stimulation for brain development prior to the onset of sensory driven excitation and, indeed, early chronic REM sleep deprivation leads to a variety of anatomical, physiological and behavioral abnormalities [86,87]. The switch from highly synchronous (“phasic”) to relatively desynchronized (“tonic”) firing can be duplicated *in vitro* over periods of many hours by the application of acetylcholine or one of its agonists [14,53,88], and was utilized by us recently (unpublished results) for examining the effect of cholinomimetic activation on functional maturation in neonatal rat cortical cell cultures grown for three to six weeks on multi-electrode plates.

**Figure 8:**
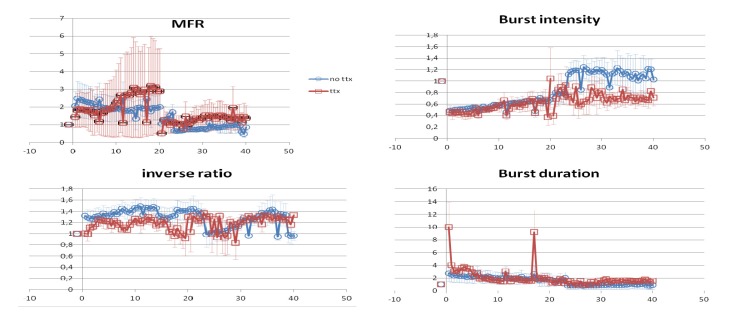
Comparison between control cultures (blue: *n* = 6) and overnight TTX (20 μM) pre-treated cultures (red: *n* = 7) for selected parameters of spontaneous activity. MFR = mean firing rate; burst intensity = intraburst firing rate; inverse ratio = the proportion of spikes falling outside bursts (interspike interval detection criterion for bursts ≤100 ms). All values were normalized with respect to the mean level in the 2-h period prior to administering 20 μM carbachol (for ~20 h), followed by ~20 h of washout. The individual means at each time point were then used to calculate a grand mean for each group, with error bars indicating the SEM. (For examples of actual polyneuronal firing patterns throughout a representative experiment, see [13]).

**Figure 9:**
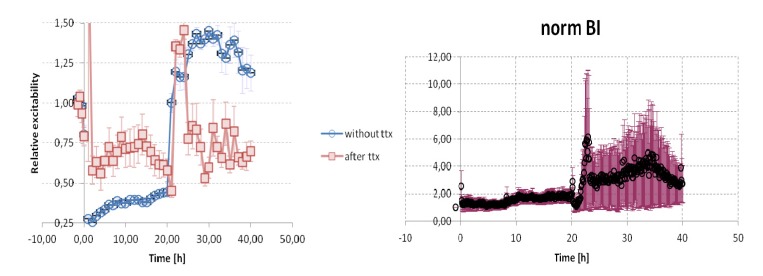
The same as in Figure 8 for two additional parameters descriing spontaneous neuronal firing in dissociated neocortical cell cultures: left—the estimated overall excitability relative to the pre-carbachol baseline is given by the “Single Pulse Response” parameter (see [90] for details); right—the Burst Index (BI: see [63] shows a strong and prolonged post-carbachol (20–40 h) augmentation in both groups, along with a very large inter-individual variance in the TTX pre-treated group.

Transfer to a carbachol containing medium almost immediately led to a strong decrease in synchronous firing [14] as well as causing the detected bursts of activity [54,55] to become significantly longer and less intense, while mean firing rates and the “inverse burst ratio” (*i.e.*, the proportion of “background” spikes falling between successive spike clusters) showed a clear cut trend towards enhancement (Figure 8). Only the inverse burst ratio was obviously affected by prior suppression of spontaneous spiking by means of tetrodotoxin (TTX), a treatment which neutralized carbachol”s subsequent acute effect (*p* < 0.05: Fisher exact-*p* test). The “burst index”, a measure of fluctuating activity levels over a period of several minutes [89], showed little or no change upon transfer to carbachol, whether or not spike discharges had been present in the preceding 24 h period (Figure 9).

Changing the interspike interval criterion for inclusion within a “burst” from ≤100 ms to ≤20 ms revealed, first of all, that “micro” bursts embedded within (and occasionally between) network bursts [41,58] were similarly desynchronized by acute addition of a cholinergic agonist to the medium: a higher proportion of spikes fell outside the detected bursts (of which there were far fewer than in control medium) while the bursts that remained were longer but correspondingly less intense in their firing (Table 1). This intra-burst smoothing of spontaneous firing could imply that cholinergic arousal of a cortical network not only reduces activity-dependent slow outward potassium currents [14] but also attenuates the inhibitory synaptic feedback responsible for repetitive synchronous bursting associated with field potentials in the “beta/gamma” frequency range [22,25,57,75]. Pre-treatment with TTX overnight, immediately prior to cholinomimetic stimulation, appeared to attenuate network responsiveness to carbachol application (Table 1) but it goes without saying that these suggestive trends will require validation with larger sample sizes (Chiappalone, M and Corner, M.A. experiments in progress).

**Table 1:** Acute and lasting effects of cholinomimetic activation in developing rat neocortex cultures.

Bursts	Rate/min	Duration (ms)	Intensity (sp/s)	Spikes-inter (%)
*Ratio of in-carbachol (first 2 h) to pre-carbachol mean values (2 h)*				
CON (6)	0.54 (*0.72*) 0.84 *	1.35 (*1.44*) 1.46 ^	0.69 (*0.70*) 0.73 ^	1.26 (*1.41*) 1.50 ^
TTX (6)	0.42 (*0.50*) 0.77 *	1.08 (*1.19*) 1.27 *	0.72 (*0.83*) 0.93 ^	1.11 (*1.20*) 1.26 ^
*Ratio of post-carbachol (first 2 h) to pre-carbachol mean values (2 h)*				
CON (6)	0.42 (*0.66*) 0.90 *	1.04 (*1.11*) 1.16	0.80 (*0.82*) 0.94	1.09 (*1.33*) 1.62
TTX (6)	0.21 (*0.31*) 0.51 ^	1.00 (*1.07*) 1.11	0.81 (*0.86*) 0.99	1.00 (*1.08*) 1.32
*Ratio of post-carbachol (last 2 h) to pre-carbachol mean values (2 h)*				
CON (3)	0.66 (*0.69*) 0.75 **	0.95 (*1.04*) 1.16	0.91 (*1.00*) 1.06	0.96 (*1.00*) 1.0
TTX (6)	0.32 (*0.42*) 0.80 ^	1.00 (*1.04*) 1.09	0.85 (*0.87*) 0.95	1.07 (*1.20*) 1.37 ^

**^** *p* < 0.02 for changes in the indicated group; * *p* < 0.02; ** *p* < 0.01 for combined changes in the two groups (Sign test, one-tailed); The numbers give the modal values (in italic type) ± the 50% range.

All parameters failed in both groups to revert to pre-carbachol values during the 2-h period immediately following return to control medium, but only the lowered incidence of detected bursts (especially in the TTX group) is as yet statistically significant (Table 1). Both groups did show, however, a strong trend towards a relatively high percentage of spikes in between the bursts. Remarkably, this was still the case in TTX pre-treated cultures more than 16 hours later (Table 1). The incidence of high-frequency bursting, along with the mean intra-burst firing intensities, also remained depressed in the TTX group even after 18 h or more in control medium. Such a long-lasting absence of post-carbachol recovery in TTX-treated cultures implies that spontaneous bioelectric activity during neocortical maturation can make all the difference between a network being able to compensate for protracted cholinergic arousal by returning to its previous levels of intrinsic neuronal bursting, or having its spontaneous activity become frozen at the cholinergically induced level .

In contrast to the marginal overall effects of cholinomimetic activation as revealed by conventional first-order measures (Figure 8; Table 1), is the application of a new quantitative measure for estimating the overall excitability of a network—the “Spike Probability Density” (SPD), a modification of the “Conditional Firing Probability [90]—which calculates the tendency for action potentials at each recording site to be followed at a given latency by a spike at another location within a specified time [91]. Not only did SPD values in all cases immediately plummet in response to carbachol (*p* < 0.01 for each group separately: Sign test), indicating a reduction of short-latency excitatory connectivity, but the extent of the decrease appeared to be attenuated in the TTX pre-treated group (Figure 9). A consistent tendency has been reported for synchronous firing to reappear after several hours and to gradually become more and more apparent over the next 20 h or so [14,88], though without ever attaining pre-carbachol values. The SPD parameter reflects this trend in the control group, whereas TTX pre-treated cultures stayed fixed at their higher plateau level for as long as they remained in the carbachol bath (Figure 9).

The build-up of synchronous burst tendencies despite continued cholinomimetic activation is also manifest as a slowly increasing intensity of intraburst firing along with a shortening of mean burst durations (Figure 8). Chronic carbachol treatment of developing cortical cell cultures over a *ca.* 24 h period has been reported to induce enhanced synapse formation and maturation, which gradually revert to baseline values upon the return of repetitive polyneuronal network discharges following washout [88]. These phenomena bear a striking resemblance to the build-up of glutamatergic synaptic excitability and delta-wave “pressure” during sleep deprivation *in vivo* [81], thus further underscoring the applicability of tissue and cell culture model systems to the identification and investigation of basic brain sleep mechanisms.

Given the preceding, it’s not surprising to see that network excitability measure (SPD) recovered in most cultures upon return to normal medium (Figure 9; *p* < 0.02: Sign test), with a few preparations even exceeding their control level. In the carbachol-only group this overall enhancement of short-latency interactions [90] was evident for up to 20 h, whereas TTX pre-treatment had the effect of limiting the SPD “rebound” to the first few hours (Figure 9). Using the original ≤100 ms. interspike interval criterion for burst definition, intra-burst firing intensities also proved to be greater in control than in TTX pre-treated cultures following carbachol washout (Figure 8). Here again, however, the large inter-individual variance in both groups makes statistical validation impossible with the small sample sizes so far available. The considerable increase in minute-order fluctuations of firing levels in both groups as a consequence of prolonged cholinomimetic activation was quite unexpected, as was the magnitude of the differences from one preparation to the next (Figure 9, BI). Such effects on “infra-slow” rhythmicity are especially intriguing in view of our current lack of knowledge about the mechanisms controlling electrophysiological fluctuations on this time scale [14,41].

## 5. Theoretical Implications

The importance of spontaneous neuronal discharges in preventing the developing neocortex from becoming hyperactive has been extensively confirmed and documented over an extensive period [69]. It might seem counter-intuitive, then, that prolonged desynchronization of firing should lead to a similar hyperexcitability in cerebral cell cultures, expressed electrophysiologically (present paper) as well as cyto-morphologically [88]. The most obvious common feature of the two treatments is the elimination of slow-wave associated burst discharges known to augment the activity-dependent entry of calcium into the neuron, the “second messenger” responsible for triggering multiple homeostatic genetic reactions [57,82,92]. This suggests that, although the “REM ontogeny hypothesis” [86] is undoubtedly correct about the importance of intrinsically generated neuronal activity for brain development, the mechanism may be more subtle than previously thought, viz., suspension during active sleep of the default neocortical slow-wave safeguards against runaway excitatory synaptogenesis. In other words, cortical maturation in homeotherms may not need so much to be stimulated as restrained, with the desynchronizing—*c.q.*, burst suppressing—effect of “arousal” during PS allowing a higher degree of intracortical excitatory connectivity to be attained than would be the case if the growth restraining actions of SWS were unopposed [14,16]. As maturation proceeds, slow-wave discharges predominate more and more during sleep, and thus would serve to prevent the definitive set-point for the balance between excitatory and inhibitory drive from becoming stabilized at too high a level for optimal brain function.

The mechanisms for establishing homeostatic set-points during ontogeny remain obscure, not in the least when they partly depend on a network that is busy monitoring its own physiological activity. Ectotherms and the most primitive endotherms [4] apparently do not require such a dense intracortical connectivity in order to function adequately, so a “forebrain connection” never needed to evolve until sometime after the initial appearance of homeothermy [93]. All this leaves unanswered, of course, the question of why PS should continue into adulthood, since each episode would—as suggested by the preliminary experiments reported above—appear to counteract the homeostatic effects of the preceding SWS episode. The answer could lie in an evolutionary appropriation of PS mechanisms for carrying out as yet unknown functions, unrelated to the optimalization of forebrain excitability for which it had originally been “designed”.

One of the important questions remaining, now that it is well established that spontaneous bursting has a significant effect on the kind of synchronous sleep-like discharges that a developing cortical network generates intrinsically once it matures [69], is whether or not the presence of such slow-wave related discharges makes any difference for the network’s ability to make the transition to a relatively desynchronized “aroused” state under the influence of cholinergic modulation (thus mimicking the natural transition from SWS to PS). Our carbachol experiments with TTX pre-treated cultures suggest that this could indeed be the case, with chronic activity-deprived preparations showing an attenuated response to cholinomimetic stimulation, both acutely and for many hours afterwards. Since the implication here is that undisturbed SWS in the intact developing animal may be a prerequisite for adequate cortical arousal during PS—and perhaps during waking as well—the first order of business now should be to verify the present results in a more extensive series of experiments (that, in fact, are now being carried out in the laboratory of Michela Chiappalone at the Italian Institute of Technology, Genoa, Italy).

The faithful preservation in reduced networks of so many of its defining neurophysiological characteristics should guarantee the continued fruitfulness of *in vitro* studies for exploring the underlying cellular and molecular mechanisms of sleep. Conversely, appreciation of the sleep-like nature of intrinsic bioelectric activity in cultured brain tissues could improve the many current attempts throughout the world to use such preparations for studying the acquisition and consolidation of externally supplied information. It is already well-known, for instance, that synchronous slow-wave activity degrades a cortical network’s activity-dependent plasticity [89], yet little attempt seems to be being made at the moment to routinely desynchronize such networks in order to carry out learning studies in a more physiologically natural manner.

Cholinomimetic arousal, as described and referred to above, might partly mitigate the “inertia” inherent in default mode—*i.e.*, isolated unstimulated-networks but, since this approach would be mimicking PS rather than wakefulness, perhaps one ought not be overly optimistic about its chances of success. Treatment with a cocktail of modulatory arousal agents [53] during the “acquisition” phase, on the other hand, followed by a permissive return to slow-wave bursting during an immediately following “consolidation” phase, would seem to be the most promising approach to activity-dependent neuroplasticity on the basis of what we presently know about state dependent learning in intact animals [81,94]. Co-culture with appropriate brainstem regions might also be considered: modest success in producing sporadic spontaneous “arousal” episodes has indeed been reported in preliminary “Frankenstein” experiments of this type [69].

Finally, at the brainstem level itself, the presence of a paroxysmally poised embedded network persisting throughout life as a relic of primitive functional organization [85] might provide some clues to the etiology of the spectacular clinical condition known as “REM-sleep Behavior Syndrome” [95]. In particular, the homeostatic nature of many early ontogenetic manifestations of neuroplasticity suggest that a deficiency in spontaneous bursting could lead to a compensatory up-regulation that, if it occurs during a critical period of development, becomes stabilized as a latent pathological alternative set-point for motoric manifestations of sleep in later life [87].
